# Frontiers in the pathogenesis of Alzheimer’s disease

**Published:** 2009-01

**Authors:** Kumar Sambamurti, K. S. Jagannatha Rao, Miguel A. Pappolla

**Affiliations:** Department of Neurosciences, Medical University of South Carolina, 173 Ashley Avenue, BSB 403 Charleston, SC 29425; 1 Department of Biochemistry and Nutrition, Central Food Technological Research Institute, Mysore - 570020, India

**Keywords:** Alzheimer’s disease, amyloid, neurofibrillary tangles, protein turnover, secretases, neurodegeneration

## Abstract

Alzheimer’s disease (AD) is characterized by progressive dementia and brain deposits of the amyloid β protein (Aβ) as senile plaques and the microtubule-associated protein, Tau, as neurofibrillary tangles (NFT). The current treatment of AD is limited to drugs that attempt to correct deficits in the cholinergic pathway or glutamate toxicity. These drugs show some improvement over a short period of time but the disease ultimately requires treatment to prevent and stop the neurodegeneration that affects multiple pathways. The currently favored hypothesis is that Aβ aggregates to toxic forms that induce neurodegeneration. Drugs that reduce Aβ successfully treat transgenic mouse models of AD, but the most promising anti-Aβ vaccination approach did not successfully treat AD in a clinical trial. These studies suggest that AD pathogenesis is a complex phenomenon and requires a more broad-based approach to identify mechanisms of neurodegeneration. Multiple hypotheses have been proposed and the field is ready for a new generation of ideas to develop early diagnostic approaches and develop successful treatment plans.

## INTRODUCTION

Alzheimer’s disease (AD) is the most common cause of dementia among the elderly. AD currently affects 12 million people worldwide (4.5 million in America) and this number is likely to triple with the aging of the baby-boom generation by 2050.[1–3] The prevalence rate for AD is about 7% for individuals aged 65 or more, and the risk doubles every 5 years after age 65.[4]

Clinical diagnosis of AD is carried out by establishing the existence of progressive dementia and then ruling out other conditions such as depression, vascular dementia, etc. The diagnosis is normally confirmed in the post-mortem brain by the presence of characteristic lesions - senile plaques (SP) and neurofibrillary tangles (NFT). The National Institute on Aging (NIA) has listed seven early warning signs of AD that are paraphrased below ( http://www.nia.nih.gov/Alzheimers/Publications/sevensigns.htm):

1. Asking the same question over and over again.
2. Constantly repeating the same story, word for word.
3. Forgetting activities that were routine, such as cooking or playing cards.
4. Losing one’s ability to manage critical activities such as managing finances and paying bills.
5. Getting lost in familiar surroundings, or misplacing household objects.
6. Neglecting personal hygiene while insisting that they were clean.
7. Relying on someone else to decide and respond on issues they normally handled themselves.

In early stages, the disease is characterized by amnesia (memory loss), visuospatial deficits, and slurred speech patterns. As AD progresses, patients display a number of abnormal and socially inappropriate behavior traits that lead to considerable embarrassment. Eventually, the disease leads to extensive disruption of activities of daily living and the patient becomes confined to nursing home care and often needs to be restrained. The patient forgets the caregiver even when they have been close for very long periods of time and ultimately even loses the sense of self. Although AD ultimately leads to death, the process is extremely slow and can last as long as 10 years. In general, early cognitive symptoms can last for up to 3 years and progress to functional dependence and behavior deficits between 1.5 and 4 years and death between 3 and 10 years.[5]

AD was initially described in the early 1900s by Alois Alzheimer as an unusual psychiatric disorder in a 51 yr old woman admitted to an asylum with cognitive and language deficits, auditory hallucinations, delusions, paranoia, and aggressive behavior. He went on to characterize the brain at the microscopic level using a number of histochemical stains that were newly developed during this period. The lesions -SP and NFT- continue to remain the main landmarks of the disease named after its discoverer. The discovery was largely ignored until Blessed and Tomlinson demonstrated the presence of AD neuropathology among the elderly with dementia in the 1960s.[6–8] However, SP and NFT fitting neuropathological criteria for AD have been recently described in the post-mortem brain of a large percentage of cognitively normal elderly individuals (demonstrated by psychometric evaluation) raising the question of whether the pathology simply marks the aging brain independent of the disease.[9] However, the lack of senile dementia in the absence of these lesions support the argument that the lesions represent an early pre-AD condition that has not yet led to progressive dementia.[10] The gross pathology of AD is also associated with considerable atrophy, particularly in the entorhinal cortex and the hippocampus. The external surface of the brain shows widened sulci and narrowed gyri, mostly over the frontal and parietal regions and ventricular dilation resulting from loss of cortex.[11,12] Early studies found that neuronal loss was associated with AD but the extent of synaptic loss was even greater. Moreover, synaptophysin-immunoreactive presynaptic terminals correlated closely with cognition.[13] In more recent positron emission tomography (PET) imaging studies, reduced glucose metabolism has been characteristically found in the medial and temporal lobe early in the disease and may predict cognitive decline in the elderly.[14]

The first major breakthrough in AD drug development was the finding that basal forebrain cholinergic neurons degenerate early in the disease.[15–17] Since the cholinergic system was found to be important for memory in several models, this finding led to the development of acetylcholinesterase inhibitors (AChEIs) for the treatment of AD. Indeed, AChEIs remain the only approved drugs for mild to moderate AD with memantine, an NMDA antagonist, prescribed for severe AD.[18] Although these treatments help to improve some of the symptoms arising from hypo- or hyperactivity of specific neuronal subtypes, the complexity of signaling in the brain and the extensive neurodegeneration associated with severe AD precludes the effective treatment with symptomatic therapy alone. The need of the day has been to understand the mechanisms underlying synaptic loss, neuronal loss, and cognitive failure to find effective ways to treat the disease. The best windows available for this type of study are to characterize the lesions of AD-namely SP and NFT- to gain a better understanding of the disease. Extensive research in this direction has led to a large increase in our information of the origin, development, and toxicity of these lesions but further study is required to reap the benefits of this knowledge.

The next major advances came with the identification of the key ingredients of SP and NFT. Early studies indicated that SP and NFT load were both correlated with dementia, but NFT’s correlation was stronger than SP.[19] Further, when SP load was corrected for NFT load, the correlation between SP and cognitive decline was lost. These studies resulted in a hypothesis that NFT may be the cause of dementia in AD and SP was only an incidental lesion. Indeed, a group of low SP and high NFT demetia of the Alzheimer type has been described.[20]

The structure of NFT was elucidated in the mid 1980s after its purification from the AD brain as paired helical filamentous aggregates of the microtubule-associated protein-Tau.[21,22] Tauopathy is also seen in about 20 neurodegenerative diseases as frontotemporal dementia, Pick’s disease (PiD), primary progressive aphasia, progressive supranuclear palsy (PSP), corticobasal degeneration (CBD), frontotemporal dementia (FTD), Niemann Pick-C (NPC) Creutzfeldt-Jakob disease, and dementia pugilistica.[23,24] Moreover mutations in the Tau gene are linked to FTD and associated with NFT formation which do not generally induce SP.[23] The availability of mouse models of FTD expressing mutant Tau has been useful in showing tangle formation and loss of neuronal function.[25] The field of taupathy has also expanded with studies showing that aluminum maltol can cause tangle formation.[26,27] Despite the presence of several models, the mechanisms that lead to tangle formation by Tau protein deposition remains poorly understood. Identification of the pathways of NFT formation is an important therapeutic target.

Although SP is less well correlated with cognition, it has been the major focus of most AD research and treated as the early lesion that triggers the cascade of reactions leading to NFT formation and neurodegeneration. The dense-core SP are usually associated with a halo of dystrophic neuritis.[28,29] Plaques are observed in layers II and III of the neocortex, dentate gyrus of the hippocampus and amygdala of the AD brain.[30] Limbic and association cortices are more susceptible than primary sensory cortex.[31,32] The core of the SP consisted of extracellular deposits of a 4 kDa peptide termed amyloid β protein (Aβ).[33,34] The peptide was found in the core of extracellular senile plaques as well as around blood vessels as cerebrovascular amyloid. Other minor components were also discovered in plaques such as alpha-1-antichymotrypsin,[35] apolipoprotein E (ApoE),[36] advanced glycation end products,[37] protein kinase C (PKC),[38] fibroblast growth factor,[39] complement,[40] and heparin sulphate glycans.[41] Some of the minor components of the SP such as ApoE have been independently implicated in the pathogenesis of AD.

The discovery of Aβ42 as the major component of the senile plaque was quickly followed by the identification of Aβ protein precursor (AβPP) and its further characterization as a large type-I integral membrane protein with a large ectodomain, a single transmembrane domain and a short cytoplasmic tail.[42–44] The Aβ42 sequence consists of a hydrophilic ectodomain (28 aa) and hydrophobic residues (14 aa) embedded in the membrane. When stored as an aqueous solution, the peptide readily self-aggregates to form oligomers and subsequently fibrils with a β-sheet structure. Monomers and fibrils of Aβ are not very toxic to neurons, but the soluble oligomers are neurotoxic in culture leading to the hypothesis that they are responsible for the neurodegeneration in AD.[45] The major support for a role of Aβ42 in AD came from the discovery of a small number of families with familial AD (FAD) linked to the AβPP gene. These mutations either increased the levels of Aβ42 or altered the structure of Aβ42 sequence to facilitate its aggregation and deposition. The families show the entire pathology seen in typical late onset AD, including the formation of SP and NFT leading to the hypothesis that Aβ42 triggers the entire cascade of pathology in AD.[44]

The next major questions are the pathways by which Aβ is produced and cleared and the subsequent mechanisms that lead to neurodegeneration. The first question has been the subject of major investigation and has discovered that AβPP is processed by a novel membrane bound aspartyl protease named BACE-1 or memapsin-2 to generate a secreted fragment - sAPPβ - and a C-terminal membrane-bound fragment - CTFβ of 99 aa.[44] A second membrane-bound protease named γ-secretase processes CTFβ to Aβ and CTFγ of 50 residues (a.k.a AICD).[46] Most secreted Aβ is 40 aa long and Aβ42 normally accounts for ~10% of total Aβ. Most of the FAD mutations on AβPP selectively increase Aβ42/Aβ40 ratios by affecting g-secretase cleavage. However, one exception is a mutation on the N-terminal side of the Aβ sequence replacing KM with NL (AβPP670NL), which increases both Aβ40 and Aβ42 as it is a very good substrate for BACE-1.[45] Most AβPP (90%) is processed by an alternative pathway known as a-secretase between residues 16 and 17 of the Aβ sequence to secrete the ectodomain-derived fragment - sAPPα - leaving behind a membrane bound fragment of 83 aa (CTFα), which is cleaved by γ-secretase to a 3 kDα fragment (P3) and CTFγ.[44–47] In addition to mutations in AbPP, FAD mutations are detected in presenilins (PS) 1 and 2, which have been identified as the catalytic subunits of γ-secretase. A number of studies on γ-secretase has determined that it is responsible for the proteolytic processing of a number of substrates acting like a proteosome of the membrane.[48–50] Some of these substrates include the developmental regulator, Notch, and cholesterol transporters - low-density lipoprotein (LDL) receptor (LDLR) and related members of this family. Contrary to simple logic, several inhibitors of γ-secretase, including antisense RNA against PS1, actually increase Aβ42 suggesting that FAD mutations may lead to an inhibition of γ-secretase activity.[48] A major advance came with the generation of mice expressing human AβPP that deposited amyloid in SP with neuritic processes.[51,52] PS1 mutations speed up amyloid deposition and increase amyloid load.[53,54] Triple transgenic mice expressing PS1, AβPP and Tau have been useful in understanding the relationship between Aβ and tangle formation.[55] Although these transgenic mice display behavior deficits, they fail to show the progressive neurogeneration characteristic of AD with amyloid-induced NFT formation, limiting our ability to logically dissect this process.[56] Moreover, numerous treatment paradigms appear to cure the behavior deficits in mice, but even vaccination, a method that eliminates SP, fails to improve cognition in human clinical trials suggesting that AD has problems beyond that detected in the mice.[57–60]

In addition to members of the APP processing pathways, ApoE alleles have been identified as major risk factors for AD. Speicifcally, it has been demonstrated that the ε4 allele increases the risk relative of AD to the more common ε3 allele.[61–63] Contrary to expectation, ApoE alleles did not affect Aβ production and early studies concluded that this protein chaperoned the aggregation and deposition of Aβ42.[64] Indeed, the finding that AβPP transgenic mice lacking ApoE fail to deposit SP and ApoE e4 mice show increase in deposition supports this hypothesis.[65,66] Nevertheless, with the typical complexity in biological systems, recent studies suggest that ApoE plays a role in Aβ degradation via neprilysin, a process that is impaired in the ε4 allele.[67] The association with ApoE is also complicated by the presence of toxic fragments of the protein, particularly for the ε4 allele.[68,69] These studies have also proposed that ApoE is responsible for the degeneration with Aβ acting as a trigger.

The finding that ApoE, a cholesterol transporter, is the largest risk factor in typical AD raises the possibility that cholesterol will play a role in AD pathogenesis. The association of other cholesterol transport and metabolism related genes (e.g., ABCA1, CYP46, SORLA) with AD support this hypothesis.[70–73] Moreover, a high cholesterol diet fosters amyloid deposition in AbPP-PS1 transgenic mice and appears to increase cleavage of APP by BACE-1.[74] Anti-cholesterol medication (statins) use appears to be protective against AD in clinical surveys although it has not been effective in AD treatment. Recent studies have also found that anti-cholesterol statins based on inhibition of mevalonate synthesis also affect AβPP processing via isoprenoids rather than cholesterol. Our studies show that isoprenoids stimulate the assembly of g-secretase and preferentially increase the production of Ab42 like FAD mutations on PS1.[75] The role of these pathways and their changes in AD need to be evaluated further.

Other causes for AD have been proposed such as oxidative stress, failure of mitochondrial function, insulin resistance in the brain, neurotrophic support failure and hormonal imbalance, copper, iron and zinc imbalance, etc.[76,77] A number of these studies have been supported by studies in animal models. However, the studies in humans have been limited by our failure to identify early stages of the disease and understand the entire metabolic process that fails in the disease. We have proposed that the failure of membrane protein homeostasis is the cause of AD. This model can explain the FAD mutations and also help in explaining the complexity of the disease with multiple system failure.[48] Animal models are limited by the smaller and less complex brain, which may allow the tolerance of higher levels of amyloid deposits and failure of other membrane protein turnover than humans. Thus, the only clear model for the disease at present are humans highlighting the urgent need for radical thinking in the development of novel disease models. The role of amyloid in diseases other than AD such as age related macular degeneration may provide valuable new directions as Aβ and ApoE seem to be deposited in this disease but the ApoE ε4 allele is actually protective against this disease. These approaches may lead to identification of drugs that may be useful for the treatment of AD and other neurodegenerative diseases.
